# Diagnostic tardif d'une hyperoxalurie primitive au stade d'insuffisance rénale chronique terminale avec hypoparathyroïdie sévère

**DOI:** 10.11604/pamj.2014.17.297.3076

**Published:** 2014-04-18

**Authors:** Zineb El Ghali, Zineb Ait Lahcen, Wafaa Fadili, Abderrahim Idrissi Kaitouni, Mohamed Hakkou, Abderrachid Hamdaoui, Inass Laouad

**Affiliations:** 1 Service de néphrologie – hémodialyse – transplantation rénale, hôpital ibn Tofeil, CHU Mohammed VI, Marrakech, Maroc; 2 Centre d'hémodialyse, secteur libéral, Maroc; 3 Laboratoire d'anatomopathologie, secteur libéral, Maroc

**Keywords:** Hyperoxalurie, insuffisance rénale chronique terminale, douleurs osseuses, anémie, hyperoxaluria, end stage chronic renal disease, bone pain, anemia

## Abstract

L'hyperoxalurie primitive est une anomalie métabolique congénitale rare caractérisée par un excès de production avec accumulation d'oxalate secondaire à un déficit enzymatique hépatique. Nous rapportons le cas d'un patient de 18 ans qui avait comme antécédent des lithiases rénales récidivantes depuis l'enfance évoluant vers l'insuffisance rénale chronique terminale, mis en hémodialyse périodique depuis 4 ans avec apparition d'une anémie résistante à l’érythropoïétine pour laquelle il a été multitransfusé et qui était admis pour prise en charge de douleurs osseuses invalidantes d'aggravation progressive associées à un syndrome tumoral fait d'adénopathies périphériques et de splénomégalie avec dyspnée et altération de l’état général. Le bilan réalisé avait objectivé de multiples images lytiques lacunaires et condensantes au niveau des poignets et des mains avec des petits reins calcifiés, une pleurésie avec péricardite de grande abondance drainée, une ascite de moyenne abondance avec splénomégalie homogène, une anémie normochrome normocytaire à 7.2 g/dl avec hyperferritinémie à 1129 μg/l et un syndrome inflammatoire biologique. La calcémie était spontanément normale à 97 mg/l avec hyperphosphorémie à 83 mg/l et hypoparathyroïdie à 74,85 pg/ml. Les PAL étaient à 136 UI/l, l'aluminium sérique à 13μg/l et la Vitamine D native à 20,87ng/ml. Le diagnostic d'hyperoxalurie a été retenu sur les données de la biopsie ostéomédullaire objectivant des dépôts de cristaux d'oxalate de calcium avec fibrose médullaire et réaction macrophagique à corps étranger. L’évolution a été marquée par la survenue de fractures spontanées récidivantes au niveau de l’épaule et des 2 hanches.

## Introduction

L'hyperoxalurie primitive est une anomalie congénitale du métabolisme responsable d'une surproduction endogène d'oxalate liée à un déficit enzymatique hépatique. C'est une maladie autosomique récessive rare dont les manifestations cliniques sont les conséquences de dépôt de cristaux d'oxalate de calcium insolubles en excès au niveau du rein et des tissus. Le diagnostic est souvent posé à un stade avancé avec des conséquences fatales.

## Patient et observation

Nous rapportons le cas d'un jeune garçon de 18 ans, 3ème d'une fratrie de 5, issu d'un mariage non consanguin, référé d'un centre d'hémodialyse privé pour prise en charge de douleurs osseuses avec altération de l’état général et syndrome tumoral. Le patient avait un long passé de lithiases rénales récidivantes depuis l’âge de 6 ans traitées par lithotripsie à plusieurs reprises sans bilan étiologique. Il a été mis en hémodialyse en 2008 pour IRCT sur néphropathie interstitielle d'origine lithiasique. L’évolution en hémodialyse a été marquée par l'apparition d'une anémie résistante à l’érythropoïétine pour laquelle il a été multitransfusé. 6 mois avant son admission, le malade avait présenté des douleurs osseuses des membres et du rachis d'installation progressive avec arthralgies inflammatoires des épaules, coudes, poignets et genoux. La symptomatologie s'est aggravée 2 mois avant l'hospitalisation par accentuation des douleurs devenues permanentes, aggravées lors des séances d'hémodialyse, entrainant une impotence fonctionnelle avec apparition de douleurs thoraciques et d'une dyspnée de repos sans toux ni hémoptysie évoluant dans un contexte de sensations fébriles et d'altération profonde de l’état général. Un traitement antibacillaire d’épreuve avait été démarré par le néphrologue référant après bilan radiologique pour tuberculose pleuropulmonaire arrêté après un mois devant la non amélioration.

A l'admission, le patient était conscient, altéré, pâle, fébrile à 38°C, tachycarde à 140 battements/min, hypertendu à 160/90 mmHg avec des oedèmes blancs des 2 membres inférieurs. L'examen ostéoarticulaire avait objectivé une douleur à la palpation des différents segments osseux au niveau des membres, côtes et épineuses rachidiennes. L'examen cardiovasculaire avait mis en évidence une diminution des bruits du cœur à l'auscultation et l'examen pleuropulmonaire un syndrome d’épanchement liquidien basithoracique gauche. Une splénomégalie homogène a été retrouvée à l'examen abdominal avec une matité à la percussion et l'examen des aires ganglionnaires avait mis en évidence une adénopathie axillaire gauche de 2cm/1cm ferme mobile avec de multiples ganglions lenticulaires au niveau jugulaire et inguinal.

**Figure 1:**
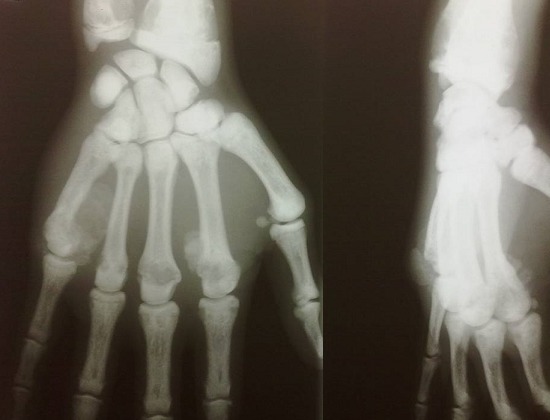
Radiographie des mains prenant les poignets: multiples images lytiques et condensantes

**Figure 2:**
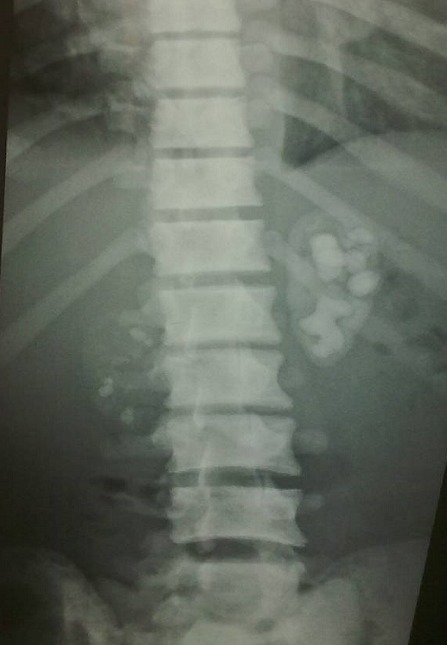
AUSP image de néphrocalcinose bilatérale avec réduits de taille

Le bilan radiologique avait objectivé de multiples images lytiques lacunaires et condensantes au niveau des poignets et des mains (Figure 1) avec une cardiomégalie associée à une pleurésie et des petits reins calcifiés à l'AUSP (Figure 2). L’échographie et la TDM abdominales avaient mis en évidence une ascite de moyenne abondance avec splénomégalie homogène sans adénopathies profondes. L’échocardiographie avait retrouvé une hypertrophie ventriculaire gauche concentrique avec hypokinésie septale, dysfonction légère du VG (fraction d’éjection à 43%), forte HTAp (72 mmHg) et épanchement péricardique circonférentiel avec invagination de l'oreillette droite ce qui avait nécessité un drainage chirurgical en urgence qui avait ramené 1 litre de liquide jaune citrin transsudatif à l’étude chimique.

Sur le plan biologique, notre malade avait une anémie normochrome normocytaire à 7.2 g/dl, avec frottis sans particularité. La ferritine était à 1129 μg/l et les LDH à 426 UI/L. Cette anémie était résistante à l’érythropoïétine ce qui avait nécessité des transfusions multiples, avec un syndrome inflammatoire: VS à 130mm et augmentation des alpha 2 globulines à l’électrophorèse des protéines sériques. Le bilan phosphocalcique avait objectivé une calcémie spontanément normale à 97 mg/l avec hyperphosphorémie à 83 mg/l et hypoparathyroïdie à 74,85 pg/ml. Les PAL étaient à 136 UI/L, l'aluminium sérique à 13μg/l et la Vitamine D native à 20,87 ng/ml.

**Figure 3:**
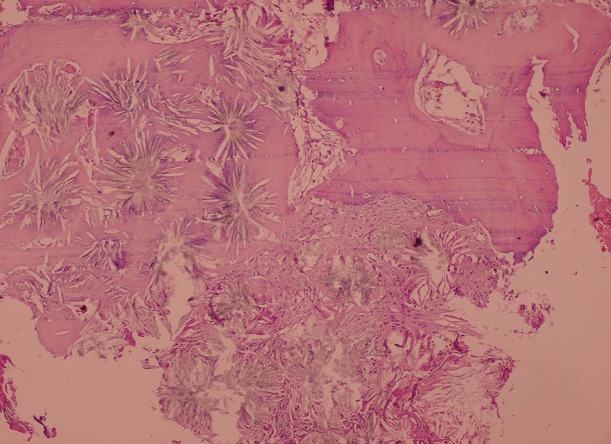
Biopsie ostéomédullaire: cristaux d'oxalate de calcium déposés en rosettes ou en étoiles

**Figure 4:**
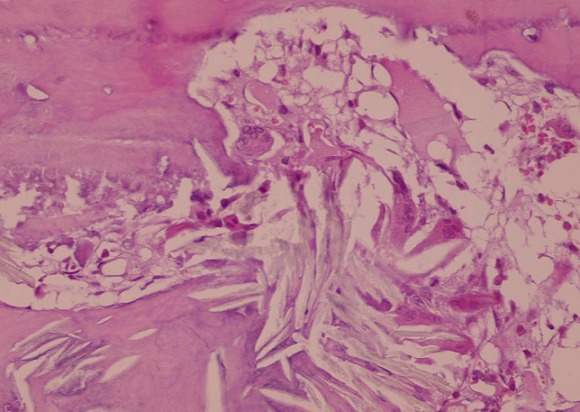
Biopsie ostéomédullaire, fort grossissement; Dépôts de cristaux d'oxalate de calcium avec réaction macrophagique à corps étranger

Une biopsie ganglionnaire a été faite au niveau axillaire et qui avait objectivé une adénite chronique réactionnelle, la biopsie péricardique avait mis en évidence un péricarde dystrophique sans signes de malignité et la biopsie ostéomédullaire était en faveur d'une hyperoxalurie avec dépôts de cristaux d'oxalate de calcium, fibrose médullaire et réaction macrophagique à corps étranger (Figure 3, Figure 4). L’évolution a été marquée par la survenue de fractures spontanées à répétition au niveau de l’épaule (Figure 5) et des 2 hanches traitées d'un côté par prothèse.

## Discussion

L'hyperoxalurie primitive est une pathologie rare dont l'incidence est estimée à moins de1 cas / million d'habitants/ an [1]. Il s'agit d'une anomalie congénitale du métabolisme hépatique entrainant une surproduction d'oxalate avec excès de son élimination urinaire. 3 formes ont été décrites dans la littérature correspondant chacune à un déficit enzymatique particulier mais toutes les trois de transmission autosomique récessive. L'hyperoxalurie primitive de type 1 étant la forme la plus fréquente [2].

La présentation clinique chez notre patient était semblable aux données de la littérature: lithiases urinaires récidivantes depuis le jeune âge avec néphrocalcinose [3–5]. Cependant, le diagnostic a été posé tardivement au stade d'IRCT après 4 ans de mise en hémodialyse avec oxalose systémique.

Les douleurs osseuses invalidantes aggravées par l'ultrafiltration et donc limitant cette dernière ce qui a aboutit à la surcharge (HTA, œdèmes, ascite et pleuropéricardite) étaient le principal signe d'appel, associées à un syndrome tumoral fait de splénomégalie et d'adénopathies avec altération profonde de l’état général ce qui avait motivé la réalisation de multiples biopsies (péricardique, ganglionnaire et ostéomedullaire) en vu d’éliminer une tuberculose ou une hémopathie maligne et c’était la biopsie ostéomédullaire qui avait permis de confirmer le diagnostic de la néphropathie initiale de notre patient après 4 ans de mise en hémodialyse en objectivant les dépôts de cristaux d'oxalate au niveau osseux avec fibrose médullaire expliquant l'anémie résistante à l’érythropoïétine. Les lésions osseuses observées au cours de l'oxalose sont liées à la réaction inflammatoire induite par les cristaux d´oxalate qui provoque la résorption osseuse. Des fractures peuvent se produire spontanément dans ces zones de faiblesse [6]. Certains signes radiologiques sont caractéristiques de l'oxalose: bandes métaphysaires denses, ostéocondensation des vertèbres. Cependant, il est en pratique difficile de faire la part entre les lésions liées à l'oxalose et celles induites par l'hyperparathyroïdie chez les malades en IRCT. La résorption osseuse semble être le résultat des deux [6, 7]. Notre patient avait par contre une hypoparathyroïdie qui semble être le facteur aggravant des lésions osseuses. Un cas similaire d'hypoparathyroïdie sur oxalose systémique a été rapporté chez un patient de 47 ans chez qui le diagnostic d'hyperoxalurie a été posé après 6 ans de mise en hémodialyse [8].

La tendance spontanée à la normocalcémie observée dans notre cas serait liée à la stimulation de l'activité ostéoclastique par les macrophages constituant les granulomes.

Sur le plan thérapeutique, théoriquement, plusieurs mesures sont proposées: hyperhydratation mais qui reste difficile au stade d'insuffisance rénale chronique vu le risque de surcharge hydrosodée et d’œdème aigu du poumon, inhibiteurs de cristallisation, administration de pyridoxine, mais la double transplantation hépatorénale reste le traitement de choix notamment au stade d'insuffisance rénale terminale [2]. Cette option thérapeutique n’était pas possible dans notre contexte.

## Conclusion

Notre observation illustre les conséquences fatales liées au retard du diagnostic de l'oxalurie primitive en l'absence d'une possibilité de transplantation hépatorénale qui reste le seul traitement efficace de cette pathologie.
